# AASLD Practice Guidance on the Use of TIPS, Variceal Embolization, and Retrograde Transvenous Obliteration in the Management of Variceal Hemorrhage

**DOI:** 10.1007/s00270-023-03654-0

**Published:** 2024-02-09

**Authors:** Dong Il Gwon

**Affiliations:** Department of Radiology and Research Institute of Radiology, Asan Medical Center, University of Ulsan College of Medicine, 88 Olympic-ro 43-gil, Songpa-gu, Seoul, 05505 Republic of Korea

Recently published AASLD (American Association for the Study of Liver Diseases) practice guidance, which was developed by consensus of an expert panel is a comprehensive one on the use of interventional radiology endovascular techniques in the management of hemorrhage from esophageal, gastrofundal, and ectopic varices [1]. This guidance addresses the recent advancements in the interventional procedures including transjugular intrahepatic portosystemic shunt (TIPS), variceal embolization, and retrograde transvenous obliteration (RTO). Moreover, this document reviews the anatomy of portosystemic collaterals and clinical use of these endovascular treatments. Although TIPS is a common procedure for variceal hemorrhage, I would like to address on the RTO procedures in this commentary. RTO is a promising treatment for the prevention of recurrent hepatic encephalopathy (HE) and bleeding of gastric varices (GVs). Furthermore, RTO may help restore portal blood flow to improve the liver function and survival in patients with cirrhosis and preserved liver function. Plug-assisted retrograde transvenous obliteration (PARTO) has been widely performed since 10 years ago, which uses of vascular plug and gelfoam [2]. PARTO deceases the procedure time and prevents procedure-related complications as it does not require an indwelling balloon catheter and sclerosing agents. Except embolization of prominent efferent vein using microcoils in a few cases, the procedure time could be markedly shortened because hand-cut gelfoam sponges (1–2 mm) themselves is used, and their larger size is usually sufficient to embolize efferent veins such as left inferior phrenic and paravertebral veins during shunt embolization. It also eliminates the need of sclerosing agents, thereby avoiding its complications. Not only has this advance been associated with high technical and clinical success, but the method is also simper, easier, and safer to perform. Although limited by a lack of clinical data, PARTO has the potential to be a treatment of choice for portosystemic shunt (PSS) associated with GVs and HE.

Patients with cirrhosis and large PSS tend to have lower portal pressure than those without shunts, indicating that they may have a certain degree of capacity to buffer increases in portal pressure after RTO. In fact, a previous study reported that an immediate increase in the hepatic venous pressure gradient of ≥ 20% above baseline was able to predict significant improvements in liver function at 6 months after RTO [3]. However, the degree of portal hypertension and the amount of residual hepatic function reserve vary greatly among patients. Abrupt increase in portal pressure following RTO may lead to the development of complications, such as esophageal variceal bleeding, intractable ascites, and hepatorenal syndrome.

Although primary prophylaxis has been well established for managing esophageal varices, no concrete guidelines are available for GVs. Previous studies indicated that in patients who have not bled from GVs and a large PSS, RTO may lower bleeding risk, though with no survival benefits. The most widely used prognostic scores such as MELD and Child–Pugh scores were shown to be predictive of clinical outcomes in cirrhotic patients treated with RTO. Recently, Choi et al. developed and validated a simple prognostic model, namely the albumin-bilirubin-international normalized ratio (INR) (ABI) score, by assigning 1 point each to three independent laboratory parameters (albumin < 3.0 g/dL, total bilirubin ≥ 1.5 mg/dL, and INR ≥ 1.5) for use in patients with cirrhosis undergoing RTO [4]. In this study, low ABI score group (0 or 1), moderate ABI score group (2), and high ABI score (3) showed significantly different survival rates. In this retrospective study, they also found that ABI score showed a better calibration function compared with MELD and Child–Pugh scores, both of which overestimated the risk of mortality or liver transplant in the high-risk population. However, further study is necessary to help physicians and patients in deciding to proceed with RTO by accurately predicting survival outcomes.
